# An international study to explore the feasibility of collecting standardised outcome data for Complex Regional Pain Syndrome: recommendations for an international clinical research registry

**DOI:** 10.1177/20494637231188333

**Published:** 2023-07-10

**Authors:** Sharon Grieve, Florian Brunner, Danylo F Cabral, Robyn Connett, Hitoshi Hirata, Norimasa Iwasaki, Yasunobu Nakagawa, Afrin Sagir, Gudson Sousa, Jean-Jacques Vatine, Nicole Vaughan-Spickers, Jijun Xu, Lisa Buckle, Candida McCabe

**Affiliations:** 1 1556 Royal United Hospitals Bath NHS Foundation Trust, Bath, UK; 2 1981 University of the West of England, Bristol, UK; 3 31031 Balgrist University Hospital, Zurich, Switzerland; 4 12235 University of Miami Miller School of Medicine, Miami, Florida, USA; 5 PPI partner, Exeter, UK; 6 12965 Nagoya University, Nagoya, Japan; 7 12810 Hokkaido University, Hokkaido, Japan; 8 2569 Cleveland Clinic, Cleveland, Ohio, USA; 9 University of Pennsylvania, Philadelphia, USA; 10 Move Rehabilitation Center, Maceió, Brazil; 11 Sackler Faculty of Medicine, Tel Aviv University, Tel Aviv, Israel; 12 Reuth Rehabilitation Hospital, Tel Aviv, Israel; 13 7423 University of Southampton, Southampton, UK

**Keywords:** Complex regional pain syndrome, pain measurement, feasibility study, international registry, core measurement set

## Abstract

**Introduction:**

Complex Regional Pain Syndrome (CRPS) is a persistent pain condition with low prevalence. Multi-centre collaborative research is needed to attain sufficient sample sizes for meaningful studies. This international observational study: (1) tested the feasibility and acceptability of collecting outcome data using an agreed core measurement set (2) tested and refined an electronic data management system to collect and manage the data.

**Methods:**

Adults with CRPS, meeting the Budapest diagnostic clinical criteria, were recruited to the study from 7 international research centres. After informed consent, a questionnaire comprising the core set outcome measures was completed: on paper at baseline (T1), and at 3 or 6 months (T2) using a paper or e-version. Participants and clinicians provided feedback on the data collection process. Clinicians completed the CRPS severity score at T1 and optionally, at T2. Ethical approval was obtained at each international centre.

**Results:**

Ninety-eight adults were recruited (female n=66; mean age 46.6 years, range 19-89), of whom 32% chose to receive the T2 questionnaire in an electronic format. Fifty-five participants completed both T1 and T2. Eighteen participants and nine clinicians provided feedback on their data collection experience.

**Conclusion:**

This study confirmed the questionnaire core outcome data are feasible and practicable to collect in clinical practice. The electronic data management system provided a robust means of collecting and managing the data across an international population. The findings have informed the final data collection tools and processes which will comprise the first international, clinical research registry and data bank for CRPS.

## Introduction

Complex Regional Pain Syndrome (CRPS) is a persistent pain condition characterised by disproportionate pain usually in a single limb and associated with sensory, motor, autonomic and trophic abnormalities. There are two types, distinguishable by the absence (Type 1) or presence (Type 2) of major nerve damage.^1^ It is categorised as an ‘orphan disease’ as fewer than 200,000 people in the United States, and fewer than 154,000 people in the European Union, are affected with CRPS each year.^2,3^ As such, the synthesis of research data is hampered by clinical studies with insufficient sample sizes to answer key questions around cause and course. Recently published revised CRPS diagnostic criteria will improve patient standardisation across studies.^4^ However, CRPS clinical trials currently use a wide range of different patient-reported outcome measures to capture the heterogeneity of the condition, which further limits pooling of results.^5^

The means to address this is twofold; (1) to promote international utilisation of a standardised set of core outcome measures and, (2) to facilitate multi-centre collaborative research to attain sufficient sample sizes for meaningful studies via the first international clinical research registry and data bank for CRPS.

Standardised, core outcome measurement sets promote the quality and comparability of research data and should be reported in all clinical studies.^6,7^ Previous consortium-led research agreed and recommended the first, standardised, questionnaire core outcome measurement set for use in CRPS clinical studies, known by the acronym COMPACT (Core Outcome Measurement set for complex regional PAin syndrome Clinical sTudies),^8^ see Table 1. More recently, an international Delphi study has defined the core *clinical* outcome measurement set.^17^

**Table 1.** Questionnaire core outcome measurement set.

Patient-reported questionnaire core outcome measurement set	Construct
Demographic data	Date of birth, gender, CRPS affected limb, limb dominance prior to CRPS, CRPS duration and participation in employment/education/voluntary work
PROMIS-29^a^	PROMIS-29 profile^9^ assesses 7 domains; physical function, pain interference and intensity, fatigue, depression, anxiety, sleep disturbance and ability to participate in social roles and activities
Suicide ideation item	Assessed using a single PROMIS item^10^
Pain intensity numeric rating scale	The least and worst pain in the previous 24 h captures the daily variability in its intensity
Short-form McGill pain Questionnaire-2 (SF-MPQ-2)	Six neuropathic items capture pain quality^11^
Pain catastrophizing scale	Impact of catastrophizing on the pain experience^12^
EQ-5D-5 L	Measurement of health state comprising mobility, self-care, usual activities, pain/discomfort, anxiety/depression^13^
Pain self-efficacy questionnaire	The respondent considers how confident they are performing each activity, while taking their pain into account^14^
CRPS symptom questions	Eight questions asking about CRPS symptoms and derived from the Budapest diagnostic criteria^15^
Patient global impression of change	To establish the efficacy of interventions in CRPS clinical studies. At follow-up only
Clinician-reported outcome measure	
CRPS severity score	Patient-reported CRPS symptoms and clinician-reported CRPS signs present on examination. A higher score indicates greater CRPS severity^16^

^a^PROMIS (Patient-Reported Outcomes Measurement Information System) is a National Institute of Health funded system, which provides validated patient-reported outcome measures that can be used in a wide range of chronic conditions.

The consortium comprises patients, clinicians, academics and industry partners from 20 countries across six continents. The COMPACT initiative meets the CRPS International Research Consortium (IRC) key objective to facilitate international collaboration and sits within its research portfolio (http://www.crpsconsortium.org).

To promote utilisation by the international CRPS research community, the combined CRPS questionnaire^8^ and clinical^17^ core outcome measurement set will be at the heart of the first international clinical research registry and data bank for CRPS. To date, CRPS research studies are often single site with small sample sizes, which limits generalisability to the wider CRPS population. There is a need for a well-characterised, international cohort study in CRPS, to improve the quality and relevance of findings. To date, there have been country-specific registries which include a UK registry recently closed to recruitment; however, this collected names and contact details but not any clinical data (31). A similar ‘address book’ is managed by the American organisation Reflex Sympathetic Dystrophy Syndrome Association (https://rsds.org/); however, the authors are not aware of any nationally co-ordinated CRPS registries in the USA. The Dutch registry led by the ‘TREND’ consortium closed over 10 years ago (https://www.trendconsortium.nl/). The authors are not aware of an existing registry that brings together the global CRPS community to collect the same clinical data. Utilising a clinical research registry methodology offers an effective and standardised way to collate a large, uniform set of prospective data from an international orphan disease population, from which it would otherwise be difficult to collate a large dataset.^18–20^ Only by establishing a consistent, international registry and data bank of this size and diversity will researchers be able to identify those factors that may precipitate CRPS, and thereby potentially develop preventative strategies. The future registry and data bank will enable researchers to request access to: (i) a large, consistent, international dataset, which will be used to gain a better understanding of the mechanisms driving CRPS, and inform targeted treatment approaches and (ii) a large, international cohort of adults with CRPS who have given their consent regarding approach for participation in research studies. The registry will answer a number of questions, with the initial study investigating the research question *‘What is the clinical presentation and course of CRPS and what factors influence it?’*

The current study was designed to test the feasibility and acceptability of collecting outcome measurement data from an international CRPS population using the agreed COMPACT questionnaire set, in order to inform an optimum protocol for the longitudinal international clinical research registry and data bank for CRPS. The findings will also inform the practicalities of collecting the defined clinical outcome data.

## Methods

### Ethical approval

The study was led and administrated from the Royal United Hospitals Bath NHS Foundation Trust, United Kingdom (UK). UK ethical approval was obtained from South Central – Hampshire A Research Ethics Committee, UK: Reference number 18/SC0322. The research team at each international research centre obtained local ethical approval. The study protocol was published^21^ and registered on ISRCTN (ISRCTN33817530) to promote transparency and reduce selective reporting.^22,23^

### Patient and public involvement

The consortium who developed the questionnaire core outcome measurement set included patient contributors from the UK, the Netherlands and Switzerland. The project management group for this feasibility study included a UK patient representative, who also contributed to this manuscript.

### Study design

Using an observational design, this international, multi-centre feasibility study aimed to:

(1) Test the feasibility and acceptability of collecting CRPS questionnaire outcome measurement data in clinical practice and according to a set protocol.^8^
(2) Test and refine the ALEA electronic data management system (EDMS) to collect and securely manage these data efficiently and accessibly, across an international population.

The key objectives of the current study are found in Table 2.

**Table 2.** Study key objectives.

	Study objectives
1	To establish and test relevant research governance, and institutional and ethical procedures required for the routine use of the core outcome measurement set
2	To test the feasibility of the recruitment process and study procedures across a representative group of future participating centres
3	To establish if data collection using the core outcome measurement set is acceptable to clinicians and patients, and to resolve any issues during the development phase
4	To establish the proportion of data that is missing from each completed questionnaire and to identify strategies to optimise data completion (target = <10% missing data)
5	To determine the optimum data collection time points to inform a future protocol
6	To determine the quantity of time required by researcher and clinician for recruitment and data collection
7	To identify a fully functioning and secure electronic data management system and to test data entry and storage processes with demonstration and feasibility study data

### ALEA data management system

The ALEA EDMS, provided by FormsVision BV and administered by a team from the Clinical Informatics Research Unit (CIRU) at the University of Southampton, UK, was used to collect and manage the study data. This comprises (1) a central bespoke registry developed by the CIRU team, and (2) an electronic Patient-Reported Outcome (ePRO) platform compatible with a computer, tablet or smart phone (https://prod.tenalea.net/ciru/ePRO/). ALEA is widely used in clinical trials internationally. The platform was compatible with the range of languages used.

### Study population and recruitment

Adults (≥18 years) with CRPS I or II, attending a face-to-face clinical visit and meeting the Budapest diagnostic clinical criteria,^15^ were recruited to the study. The broad inclusion criteria aimed to capture representation across different ages, gender, ethnicity, disease durations and registry access requirements. Only those unable to understand the written word or unable to write, and/or unable to give informed consent were excluded. Participants were recruited from seven international research centres across six countries: two in Japan, with one each in the United Kingdom, United States, Switzerland, Israel and Brazil. Each centre aimed to collect ≥10 (maximum 30) completed Time 1 (T1) and Time 2 (T2) datasets, with the two centres in Japan agreeing to collectively recruit 10 participants. These countries represented the diversity of future registry and data bank users. Recruitment was between December 2018 and September 2020, initially commencing in the UK. International sites opened to recruitment once local ethical approvals were obtained. Each site closed to recruitment when the mutually agreed recruitment target was reached or the Principal Investigator indicated recruitment capacity was achieved.

### Informed consent

Patients attending the participating study centres for a routine clinical visit were recruited at any point in their treatment pathway. A member of the multidisciplinary team provided potential participants with a recruitment pack comprising: an invitation letter, a participant information sheet, the baseline questionnaire set (see Table 1), two copies of the consent form (one for the patient’s own records) and a contact details form to enable the participant to be contacted at T2. Patient-facing study documents were provided in local languages and translated either (i) via a UK-based translation service, or (ii) by local researchers following a best practice protocol^24^[2]. At each centre, the local research team were available to answer any patient questions. Informed consent was obtained by the return of a signed and dated consent form to the local team.

### Summary of data collection

After informed consent, the following data were collected:

1. The patient-reported questionnaire set of outcome measures (Table 1) were completed at baseline and at 6 months (T2) at all sites, with the exception of Brazil, who undertook T2 at 3 months. T2 data were collected at 3 months in Brazil to compare whether a shorter timeframe improved the response rate. To capture health at a specific time point, participants were asked to complete the questionnaire during a single day if possible. Where applicable, permission was obtained from the distributors or licence holders, to use the standardised questionnaires for the purposes of this study and to supply them in the relevant languages. Where standardised translations were not available, they were provided by the local research team following a best practice protocol.^24^
2. The clinician-reported CRPS Severity Score (CSS)^16^ was completed at baseline and at T2 if the study participant attended a face-to-face clinical appointment at this time. These data were only included in the study if the participant gave explicit informed consent. The CSS is directly derived from the Budapest CRPS diagnostic criteria^15^[16]. The number of patient‐reported symptoms, and CRPS signs observed on examination by the clinician, are added to give a total. Higher scores indicate greater CRPS severity (range 0–16).^16^ The CSS was completed in English by clinicians at all sites with the exception of Brazil where it was completed in the local language.

#### Data collection at T1

All participants completed a paper version of the questionnaire at baseline (T1); the latter defined as the signatory date recorded on the consent document. It was completed at the research centre or at home and, if the latter, returned by prepaid post. At T1, participants indicated on the contact details form whether they wished to receive the T2 questionnaire on paper or electronically via ALEA ePRO.

#### Data collection at T2

At T2 (±2 weeks), participants were provided with a second questionnaire in the format they selected at T1. This collected fewer demographic data than at T1, but included a patient global impression of change.^8^

Paper versions were posted to the participant shortly before the 6‐months time point, accompanied by a letter which re-familiarised the person with the study and a prepaid envelope for return to the local study team. If a clinical appointment was scheduled at this time, the questionnaire was given by hand and the CSS was completed by the clinician. A CSS was accepted if completed within a ±2 weeks window of the 6-months time point. Participants who requested an electronic version of the questionnaire received an email approximately 2 weeks before the 6‐months time point, containing an e-link, and instructions, to access ALEA’s ePRO environment. Text preceding the questionnaire re-familiarised the participant with the study.

If the questionnaire was not completed within 14 days, one reminder letter was sent by post or email.

### The data collection experience

All participants were invited to complete a section at the end of the T2 questionnaire asking for written feedback on their experience of completing the baseline and T2 questionnaire. A series of questions asked about their experience of completing the paper version and, where applicable, completing the questionnaire via ePRO. Questions included the time taken to complete the questionnaire, and feedback on the layout, question order and completion instructions.

At each research centre, clinicians and/or researchers delivering the study were invited to provide feedback on their experience of recruitment and data collection via an e-questionnaire. This comprised questions around their experience of their local approvals process, the recruitment process and their experience of data collection including use of ALEA.

### Data analysis

We aimed to ascertain the practicalities of collecting outcome data across a range of different populations and cultures. We did not aim to derive statistically significant data. Data were collated on patient recruitment, including total number of patients recruited per centre; consent rate; participation rate and loss to follow‐up. Data recording participant preference of paper versus e-questionnaire were also collated, along with the associated response rate. These data, and key findings from the patient and clinician feedback questionnaires, were synthesised to inform the final data collection tools and processes that will comprise the first international, clinical research registry and data bank for CRPS. This included consideration of key difficulties and commonalities across study centres. Topic areas within the patient and clinician feedback data determined the acceptability of data collection processes and timings.

### Project management group

A project management group convened regularly throughout the study via teleconference to review progress and comprised: a patient representative, the study Chief Investigator, the Co-Chief Investigator, a representative from the University of Southampton CIRU and the Bath administrative team. A regular newsletter was distributed via email to each study centre reporting on recruitment and pertinent study issues. Teleconferences between the administrative team and each local recruitment centre offered additional support.

## Results

### Research governance, institutional and ethical procedures

Across the seven research centres, the mean time from submission of ethics to receipt of approval was 16.5 weeks. Japan (Nagoya) had the shortest approval time of 11 weeks, with Brazil the longest at 34 weeks. Researchers in Brazil reported delays due to the local impact of the COVID-19 pandemic. There were local challenges in seeking ethical approvals in some international centres. For example, in Brazil local *and* national approval was required, which extended the process. To mitigate the delay, T2 data collection was advanced from six to 3 months, which allowed all research centres to complete the study by March 2021 and provided an opportunity to compare T2 response rates at different time periods.

The central administration team supplied each research centre with documentation to support their regulatory processes and this was in the local language when requested.

Two substantial amendments were submitted during the study. The first obtained approval to gather participant feedback at all sites via a questionnaire rather than the originally intended focus groups. This responded to PI feedback that patient focus groups are poorly attended in Japan due to cultural preferences. The second obtained approval for participants who had not responded to the questionnaire within 14 days to be reminded via a telephone call at T2. This enabled us to better understand if there were any issues with participants receiving the questionnaire and to re-send it if necessary.

### Testing the recruitment process and study procedures

98 adults with CRPS were recruited across seven research centres (mean age of cohort 46.6 years, range 19–89 years; female *n* = 66). The characteristics of the research centres are in Table 3. In total, a minimum of 169 people were invited to participate across all centres, indicating a recruitment rate of ≤58%. Study recruitment reached the agreed target (Israel, Switzerland, UK, USA) or capacity (Brazil and Japan) at all centres. Definitive numbers of all those approached were not recorded at every centre.

**Table 3.** Research centre characteristics.

Site	Brazil	Israel	Japan	Switzerland	UK	USA
Number of patients recruited (total *n* = 98)	8	21	9	21	22	17
Response rate of those approached to participate in the study	35%	95%	75%	100%	36%	49%
Recruitment period (months)	4	11	9	10	13	11
Data collection time point at T2 (months)	3	6	6	6	6	6
Number of patients choosing a paper questionnaire at T2	7	21	9	8	12	10
Number of patients choosing e-PRO at T2	1	n/a^a^	n/a^a^	13	10	7
Number of patients completing T1 and T2 (total *n* = 55)	5	5	8	12	16	9
Study retention rate at T2 %	63%	24%	89%	57%	73%	53%

^a^Not applicable as the ePRO option was not offered.

The conversion rate of those approached to participate varied widely, ranging from >95% recruited (Switzerland and Israel) to 35% (Brazil and UK). The recruitment period ranged from 4 to 13 months for each research centre. In the UK, recruitment had closed prior to the COVID-19 global pandemic. However, all other international centres were recruiting during the pandemic, and this was reported to have affected participant recruitment at several sites. In Brazil, ethical delays resulted in a shorter recruitment period to allow for T2 data collection to be completed by study close. In the United States (US), patients were asked to provide consent at the time they agreed to receive the recruitment pack (*n* = 34); however, only 17 people returned their T1 questionnaire and were therefore included in the study.

Fifty-five patients completed both T1 and T2 documentation. Study retention rates were highest in Japan (89%) with only one participant lost to follow-up at T2.

Four research centres offered patients the choice to complete the T2 questionnaire electronically via e-PRO and 31 patients chose this option (46%). However, only 13 of these patients went on to complete it (42%). The research teams in Israel and Japan did not offer the e-PRO option at T2 due to cultural preferences. The return rate of a completed questionnaire was higher for those choosing the paper version at T2. Across all centres, 67 people chose this option and 42 returned it (63%).

### Testing the ALEA data management system

ALEA, our electronic data platform, proved a robust method of collecting and managing the data and with only 0.35% missing data, far exceeding the target of <10% missing data. The platform was compatible with all the required languages; either via upload of previously translated documents or using embedded language directly via the platform. Automated scoring of questionnaires was successfully incorporated into the platform by the CIRU administrative team. The data extraction process was tested and was able to fulfil the required capability. Only two technical issues were reported to the central administration team by patients using e-PRO; a login issue, which was quickly resolved, and a request for a paper questionnaire, as the participant was unable to complete the e-PRO version within the timeframe.

### Patient and clinician feedback

18 patients, (UK (*n* = 12), Switzerland (*n* = 2) and Brazil (*n* = 4)), provided feedback on their experience of completing the paper version of the questionnaires. All were satisfied with the appearance and question order of the paper version, and found it easy to understand. Most respondents (61%) reported completing the paper questionnaire in ≤29 min and all in a single session. Of the remainder, 28% took between 30-59 min and 11% took ≥60 min. Three participants chose to complete the questionnaire in more than one session. Four patients provided additional feedback on their use of e-PRO, which included suggesting changes to the format, such as increasing the size of the font. For ePRO, the completion time was evenly spread and ranged from <15 min to > 1 hour. Participants from Japan, Israel and the USA chose not to provide feedback on either data collection method with no reason given.

Nine clinicians provided feedback on their experience of recruitment and data collection. Issues identified included a delay in the return of the T1 questionnaire and, in some instances, it was not returned. Local postal issues were reportedly the cause of some delays. The COVID-19 pandemic was identified as an obstacle to recruitment due to national lockdowns affecting healthcare provision and resulting in delayed or cancelled patient visits. The clinician respondents reported it took 5–10 min to input the questionnaire data received from each participant, and a similar time to input the CSS data. The clinicians and researchers reported ease of ALEA data entry and any minor issues were resolved quickly with the support of the ALEA team at the University of Southampton, UK.

## Discussion

This study provides insight into the processes and practicalities required when establishing an international clinical research registry and data bank for CRPS. It was designed to inform the set up and management of the future CRPS registry; however, learning could be applied more widely when establishing a registry for other health conditions.

The study provided a platform to test governance and ethical processes across international research centres utilising different national guidelines; a scenario that will be replicated when ethical approval is obtained for the future long-term registry. Where possible, the central administrative team supported the international researchers in fulfilling the requirements of their local ethical and governance processes, for example, the provision of supporting documentation and advancing T2 data collection in Brazil to support timely study completion. The central administrative team was unable to support the local ethical approvals process directly; however, a full set of study documentation was supplied to each centre, in the local language where required, thereby facilitating the submission process. Some ethical review bodies in non-English speaking countries were willing to accept documentation in English, which accelerated the process and negated the need for additional translations.

This study was partially conducted during the COVID-19 global pandemic and this was a limiting factor for recruitment and data collection at several of the international research centres. Despite the challenges this presented, study total recruitment reached the agreed target or capacity at all centres. The response rate of those approached to participate reflected the different recruitment strategies used at the local recruitment centres. When obtaining patient consent during a face-to-face clinical consultation, the response rate increased. Conversely, when potential participants considered the study information at home, the response rate was lower. The reason for this discrepancy is unknown; however, patients may be more likely to discard the study information when at home or be more eager to please their clinician in clinic. There may also be cultural differences affecting research recruitment numbers as some centres had a very high ‘recruitment to participation’ conversion rate.^25^ Despite these data, an option for e-consent will be available for the future registry, to provide improved access choices for participants, and cost-effectiveness and reduced administration for researchers.^26^

This study highlighted the international variation in recruitment strategies for example in the United States, patients consented on receipt of the recruitment pack; however, not all went on to return the T1 questionnaire. This approach initially indicated a higher recruitment than the reality. It is not possible to conclude whether this method improved study participation or not. The future registry will allow flexibility in the recruitment process to reflect the range of strategies used internationally.

Of those recruited, 56% completed the study (T1 and T2). However, the loss of data from approximately half of recruited participants has implications for the quality of the future long-term registry outcome data. A range of retention strategies were adopted, for example, participants were offered a choice of paper or e-data collection at four sites, an approach associated with improved retention,^27^ and reminder letters and emails were also used. At some centres, it was reported national postal strikes had negatively affected the return of some paper questionnaires; however, more work is needed to optimise longitudinal data collection response rates. Clinical studies collecting patient-reported outcome data via questionnaire completion are widely reported as liable to participant attrition^28,29^ and this may lead to registry quality being threatened by incomplete data.^30^ In addition, missing data may result in study bias if attrition sits with certain study populations causing them to be under-represented.^31^ Retention strategies for the future registry will be informed from the literature,^27,32,33^ consulting with colleagues who have experience of leading registries, and, most importantly, engaging people with CRPS via online focus groups to gain the insight of the intended registry population.^34,35^ Retention strategies for consideration include offering alternative methods of data collection, regular contact with participants and developing a registry ‘community’.

Four research centres offered patients a choice of paper or electronic data collection at T2, to provide an indication of how widely each would be utilised in a future registry. Almost half of eligible participants did express a preference to complete T2 electronically via e-PRO, confirming the need for this option in the future; however, only 42% of these went on to complete it. A question within the participant feedback questionnaire attempted to elicit the reason for choosing e-PRO at T2 but this question generated no responses. The completion rate for those choosing paper at T2 was higher at 63%, despite issues around postal strikes and it was reported by participants that the paper version was chosen at T2 as it was ‘easier’ and especially for those ‘not good with electronics’.

This paper reports a pragmatic study to explore the feasibility of collecting standardised outcome data for CRPS; however, there were some limitations. The study was completed during a global pandemic and this may have negatively affected recruitment due to the restructuring of clinical services. Participant feedback was more limited than we had anticipated and other methods of collecting feedback, for example, via local focus groups, may have been more successful. Cultural considerations had influenced the protocol development, for example, researchers in Japan indicated participants may be reluctant to share feedback openly and were more likely to respond via questionnaire. In addition, following the recommendations of local researchers, the electronic version of the core outcome measurement set was not offered to participants at all sites and this may have had an impact on response rate. In some instances, the study findings did not provide sufficient insight to inform the future registry and data bank protocol, for example, the optimum time points for participants to return the completed questionnaires. In order to obtain consensus, we conducted a later online survey, to collect these data from key members of the wider COMPACT consortium. The results informed the data collection time points for the future registry (Table 4).

**Table 4.** Key recommendations.

Process	Key recommendations for the future international registry
Ethics and governance	Realistic timelines for study set up in each research centre should be agreed, after discussion with local team
	At each recruiting centre, the approvals process should be started as soon as possible
	Research centres should be given access to a complete set of study documentation in the core set of languages. A best practice translation protocol should be provided where applicable
	Centres should identify colleagues who are familiar with the local ethical process and could potentially offer support
Recruitment	The protocol should include flexibility in recruitment processes to reflect the range of strategies used internationally
	Strategies should be in place to maintain momentum for sites to recruit to the study, for example, regular newsletters to establish a registry community
Data collection	Data collection should be at baseline, with the expectation that a minimum of one follow-up time point is completed at 3, 6 and/or 12 months
	Participant email alerts and reminders should be introduced prior to the questionnaire completion dates. Participants should be sent a regular study newsletter to promote a registry community
Data management	Automated scoring of each outcome measure included in the questionnaire set should be incorporated into ALEA for the future registry
	The central administrative team should provide all participating centres with a detailed guide for accessing and using ALEA, including contact information in the event of any technical difficulties

## Recommendations for the future international clinical research registry and data bank

The key recommendations are presented in Table 4.

This study has confirmed the questionnaire core outcome measurement set is feasible and acceptable to collect, according to a set protocol, in clinical practice and across a range of populations and cultures. The ALEA EDMS provided a robust means of collecting and managing the data efficiently and accessibly across this international population. The findings will inform the establishment of the longitudinal international clinical research registry and data bank, which has long-term importance in relation to the prevention and treatment of CRPS. Once established, the registry will provide access to a large and consistent set of CRPS outcome data, which will improve our understanding of the cause, course and optimum management of CRPS.
